# Can Dental Lesions Be Foreseen and Prevented?

**Published:** 1879-02

**Authors:** 


### CAN DENTAL LESIONS BE FORESEEN AND PREVENTED?

BY DE–CE–AICH.

If dentistry had a literature like medicine and if it had an equal antiquity, then, perhaps, dentistry might at this time have been, more than it is, a system of preventing dental diseases. With praiseworthy exceptions practitioners put upon themselves little beyond the task of removing disease when it presents a well marked degree of development. Anything short of this is regarded as not requiring treatment yet. We replace lost organs and arrest the disease in wasting ones. Having thus done something to render the patient free from present suffering, we send him away with morbid action going rapidly forward in every part of the mouth to wait until some particular tissue is sufficiently wasted to warrant our interference. Very likely by way of doing our whole duty, we have told the patient in general terms to clean his teeth. This done, all parties calmly await future developments. Both dentist and patient seem to have settled down upon the conviction that caries is inevitable, and that art can only follow in the wake of the destroyer.

